# How do physicians behave when they participate in audit and feedback activities in a group with their peers?

**DOI:** 10.1186/s13012-018-0796-8

**Published:** 2018-07-31

**Authors:** Lara J. Cooke, Diane Duncan, Laura Rivera, Shawn K. Dowling, Christopher Symonds, Heather Armson

**Affiliations:** 1 Department of Clinical Neurosciences, Cumming School of Medicine, University of Calgary, UCMC Area 3, 3350 Hospital Drive NW, Calgary, AB T2N 4N1 Canada; 2 Cumming School of Medicine, University of Calgary, HSC G302, 3330 Hospital Dr NW, Calgary, AB T2N 4N1 Canada; 3 Division of Endocrinology and Metabolism, Cumming School of Medicine, University of Calgary, 3330 Hospital Dr NW, Calgary, AB T2N 4N1 Canada; 4 Department of Family Medicine, Cumming School of Medicine, University of Calgary, 3330 Hospital Dr NW, Calgary, AB T2N 4N1 Canada

**Keywords:** Audit and feedback, Feedback, Social learning theory, Practice improvement, Professional development, Physician learning, Implementation, Knowledge translation

## Abstract

**Background:**

Audit and feedback interventions may be strengthened using social interaction. With this in mind, the Calgary office of the Alberta Physician Learning Program developed a process for audit and *group* feedback for physician groups. As a part of a larger project to develop a practical approach to the design and implementation of audit and group feedback projects, we explored patterns of physician behavior during facilitated audit and group feedback sessions.

**Methods:**

Six audit and group feedback sessions were recorded, transcribed, and analyzed thematically to derive a conceptual model of physicians’ behaviors during audit and group feedback sessions.

**Results:**

A predictable cycle of behaviors emerged from audit and group feedback sessions. This cycle would repeat with discussion of each new data element: reacting to the data, questioning and understanding the data, justifying and contextualizing, sharing and reflecting on the data and relevant guidelines, and planning for change. “Change cues” that emerged within groups reliably pivoted the discussion towards action planning.

**Conclusions:**

In audit and *group* feedback sessions, physicians display a predictable series of behaviors as they move towards commitment to change. Establishing the meaning and credibility of the data is a necessary precursor to reflection. Group reflection leads to “change cues” triggered by group members, which stimulate action planning.

## Background

Audit and feedback (AF) is one widely published strategy to help physicians translate knowledge into practice. While AF outperforms many traditional continuing medical education approaches to foster physician behavior change, AF effectiveness on change in physician compliance with desired behavior varies across published intervention studies from − 9 to 70% [1, 2]. Why is this? Ivers et al. called for “no more business as usual” in AF research, citing the paucity of new contributions to the science of AF over the last two decades [3]. They highlight the failure to understand the “mechanisms of action” of AF and the lack of theoretical underpinnings in the design of AF interventions [3–6].

Brehaut et al. proposed 15 suggestions to guide AF design in an effort to improve AF effectiveness [7]. Many of these suggestions are recognizable as best practices from implementation and behavior change science, for example, choosing an appropriate “desired action” as the focus of AF and choosing clinical questions that physicians believe they can impact [7, 8]. The authors also suggested ways to deliver the feedback for best effect, such as provision of repeated “instances of feedback,” individualized data, and data with comparators [7, 9]. Brehaut et al. made recommendations on enhancing the perceived credibility of the data, on how to display feedback, and the need to address barriers to change [2, 7, 10–12].

Most directly relevant to *learning theory* was the final recommendation of Brehaut et al., to “construct feedback through social interaction” [7]. This recommendation originates from social learning theory that posits that development and learning occur through observation and interaction with others in relation to the learning material [13]. Further support is derived from the work of Vygotsky, who identified the value of having a “more knowledgeable other” present to promote learning and who contended that learning is derived from socially constructed groups [14].

More recently, authors in the area of feedback in medical education have emphasized that relational issues between teacher and learner, the perceived credibility of the individual providing the feedback, and mutual respect between the two parties play a crucial role in the uptake of feedback [15–18].

This interactive and relational component of feedback has had minimal consideration in previously published AF intervention studies, where the focus has been on the delivery of passive feedback to physicians using data report cards or one-on-one feedback. The physician interaction with the data and the relationship between the giver and receiver of feedback have not been explored in depth in the setting of AF [2].

The opportunity to explore physicians’ understanding of their data in the context of their practice has been shown to be a prerequisite to effective implementation of the feedback [15]. Further, feedback uptake is enhanced through the development of rapport, trust, and mutual respect through which a “foundation for meaningful conversation about performance assessment” can occur [15].

Uptake of feedback is also influenced by the goals or motivations of the participants involved in the learning setting. These goals have been explored in achievement goal theory (AGT) which explores the differing goals that are pursued by learners to feel competent [19, 20] and how these goals impact motivation, define success, and clarify their subsequent behavior [21]. AGT proposes that there are mastery and performance goals. Mastery goal orientation focuses on developing competence relative to their previous performance or not to lose competence (mastery avoidance goals). Performance-orientated goals still focus on performing competently relative to others or on preventing the appearance of being incompetent in comparison to other participants (performance-avoidant) [22]. Although most of the research in this area has focused on the individual, there is beginning to be more interest in the impact on the social context of learning [23]. The social context can orient participants towards a particular goal orientation (e.g., relevant tasks, sharing a group responsibility for improvement, confidential evaluation result) [23, 24].

Previous AF intervention studies have seldom reported the use of theory in the design of AF, and when present, its use was poorly described [4, 6]. A recent study reported findings from interviews with theorists from cognitive psychology, education, and medical decision-making who generated “theory-informed hypotheses about how to improve AF interventions” [5]. The experts posited several relevant hypotheses related to feedback delivery. For example, AF effectiveness will be enhanced if the participants are engaged in “social discussion about the AF”, the facilitators create learning opportunities, and the AF is provided “in-person” [5].

Taking into account medical education theory and design elements from implementation science, the Calgary office of the Alberta Physician Learning Program (CPLP) developed an approach to a novel form of audit and feedback presented within a collegial peer environment. The CPLP develops and delivers audit and *group* feedback (AGF) projects to Alberta physicians. This study implemented many of the components suggested by Brehaut et al. including clinicians’ generation and co-creation of the clinical questions, ensuring the clinical relevance, credibility, and the potential impact of the data, and the provision of both individualized data and comparators in an interactive, in-person, facilitated, group feedback session. Using the CPLP, as an AGF “laboratory,” we studied the interactional nature of AGF sessions (AGFs), which provided both facilitation and opportunities for interaction with peers to explore the data, to develop priorities, and then to develop and explore implementation strategies.

This study was focused on exploring physician behaviors during the AGF sessions and how these contributed to interaction during the session and ultimately to the discussion of change and implementation strategies by the physician groups.

Based on these findings, we present a conceptual model of physician behaviors in AGFs.

## Methods

Ethics approval for this work was received for each AGF project from the Conjoint Health Research Ethics Board: REB13-0075 (project 1); REB14-0484 (projects 2a, 2b, 2c, 2d); REB13-0459 (project 3).

### Setting

**Fig. 1:**
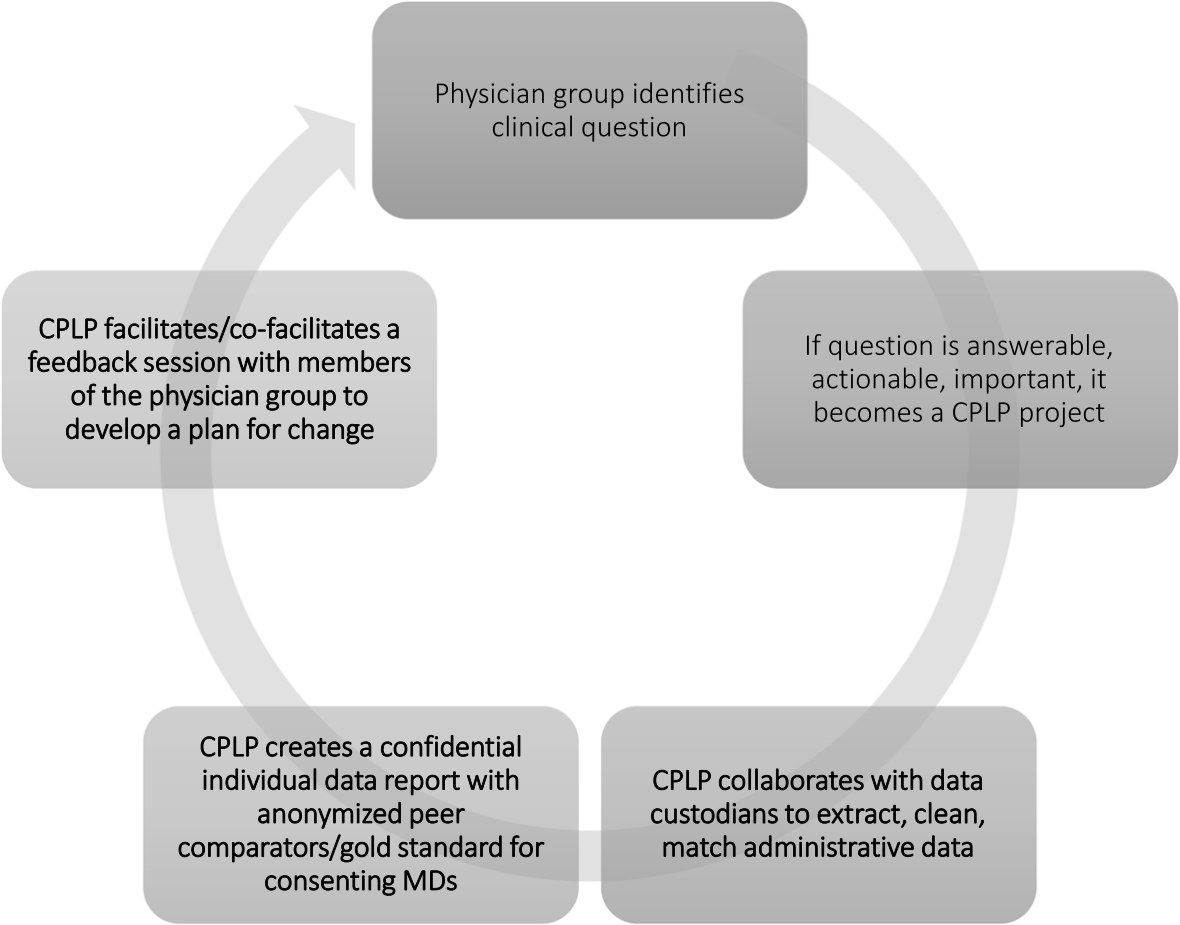
The CPLP process from clinical question to AGFs. Physician groups bring clinical questions of interest for review by the CPLP. The CPLP team reviews the questions for appropriateness for audit and feedback. Consideration is given to impact, reach, actionability, and accessibility of the data. CPLP collaborates with data custodians to make individualized AF reports for consenting doctors. The confidential reports include individual data with anonymous peer comparators and relevant best practice information. Consenting physicians then participate in a facilitated group feedback session with their peers, led by a CPLP and/or participant facilitator. As a group, the physician peers review each aggregate data point, along with their own performance reports and seek opportunities for practice improvement

This study took place at the Cumming School of Medicine at the University of Calgary and involved analysis of the work of the CPLP between 2014 and 2016. The Alberta PLP was developed in 2009 to deliver audit and feedback reports about practice patterns to Alberta physicians. As a program funded by the provincial medical association, it is available to all physicians in the province. In order to participate, a physician or physician group (such as a department, division, clinic, or care network) approaches the program with a clinical question they wish to explore. The program staff work with the individual or group of physicians to clarify the question, the perceived practice gap, and the clinical importance of the topic. If the question is appropriate for AF, a CPLP project is designed and implemented. The CPLP project culminates in a facilitated AGFs in which consenting physician participants have their individual data reports (which include anonymized peer comparison data) and work as a group with a CPLP facilitator (the medical director, who was the same individual for each of the included AGFs) to review their data to identify opportunities, barriers, and enablers for change [9, 11]. The facilitator would walk the group through the data reports item by item using an anonymized aggregate report that was projected at the front of the room while the participants would follow along with their own report. The facilitator was a physician working for the CPLP program. He was not a member of any of the physician groups receiving feedback. The facilitator’s role was to lead the group discussion during which physicians would review their individual data for each item that was audited and then discuss with the group their findings and how they might be interpreted. The facilitator’s goal for each group was to help the groups to identify practice variations that, if addressed, could improve patient care. The facilitator’s primary role in the sessions was to explain the data extraction processes, data limitations, and interpretation of the tables provided in the report to the physician group. The flow of the group discussion was dictated by the contents of the reports and the direction taken by the group participants. The CPLP process is illustrated in Fig. 1.

### Research design

In early audit and feedback sessions hosted by the CPLP, it appeared anecdotally that specific, discrete physician behaviors emerged during AGFs. In order to study this more fully, recordings were made of each of six AGFs that took place between 2015 and 2016. These were transcribed verbatim and analyzed thematically by the research team [25]. This approach facilitated the exploration of the behaviors and interactional nature of the physician groups during individual AGFs.

### Participants

All physician participants provided written informed consent to allow access to their administrative health data for purposes of creating AF reports and to record, evaluate, and study the AGFs in which they participated.

Six AGF projects were studied. Projects 1 and 3 consisted of two different specialty groups looking at various aspects of practice variation in two different hospitals. Projects 2A, B, C, and D, were a part of a de-prescribing project with a group of family physicians working as hospitalists at four different acute care sites. Although the clinical question was the same for groups 2A, B, C, and D, the groups were quite different. Each physician group practiced in a different acute care hospital and served a unique population of patients (palliative, geriatric, immigrant, high acuity, etc.) and each group was composed of physicians who only worked at that one hospital and as such had its own unique culture and dynamic.

### Project selection

For purposes of this study, a project was defined as, a project was used as the process of obtaining, visualizing, and reporting administrative health data to answer a group of physicians’ question about their practice, and culminating in an AGFs.

The projects selected for this study represent all of the AGFs that took place through the CPLP between January 2015 and January 2016. They were selected because they were being offered in relatively short succession, such that researchers could begin to understand the respective cultures, patterns, and influences at play within each group and observe behavior and outcomes as they emerged over time.

### Data collection

Our data source was verbatim transcripts of each audit and feedback session.

### Audit and group feedback session qualitative analyses

The research team used an iterative process of inductive thematic analysis of each of the AGFs transcripts. Initially, two team members (LC, DD) free-coded all six transcripts, identifying emerging themes and modifying the coding with each subsequent transcript. None of the researchers were members of the physician groups under study. Next, four members of the research team (LC, DD, LR, HA) met to discuss the identified themes and organized them into a coding framework that reflected the nature of the sessions. Two members of the team (LC, DD) re-coded all of the data according to the agreed upon coding structure and then reviewed the transcripts together to look for agreement, missing themes, or inconsistencies. Inconsistencies in coding were reviewed within the broader context of the discussion recorded in the transcripts, and a final decision about coding was reached by consensus between the two coders. The team reviewed the final coding structure together to develop a conceptual model that captures what happens in AGFs.

## Results

A total of 50 physicians participated in AGFs. Nine attended the project 1 AGFs, 28 attended project 2A, B, C, and D AGFs, and 13 attended the project 3 AGFs.

Iterative coding and analysis of the six audit and feedback transcripts revealed four main themes and seven sub-themes within which all of the qualitative data from AGF sessions could be categorized with excellent agreement between coders (LC, DD). The four main themes were (1) interpreting the data, (2) understanding evidence about best practices, (3) “change cues,” and (4) “change talk.” The majority of the qualitative data fell into the theme of “interpreting the data,” which was further categorized into four sub-themes: (i) reactions, (ii) understanding and questioning, (iii) justifying and contextualizing, and (iv) reflecting on the data. One sub-theme, “justifying and contextualizing,” and one overarching theme, “change talk,” were further categorized as shown in Table 1.

**Table 1:** Overarching themes, sub-themes, and sub-divisions of the qualitative analysis of six AGFs

Overarching themes	Sub-themes	Sub-divisions
Interpreting the data	Reactions	
	Understanding/questioning	
	Justifying/contextualizing	Data is affected by other groups/factors
		Data affected by personal factors
		Data affected by patient factors
		Data affected by system factors
	Reflecting	
Understanding evidence about best practices		
Change cues		
Change talk	Actions the group should take	
	Personal actions a group member will take	
	Actions requiring involvement of others/other groups/other professionals	

### What happened during AGF sessions?

#### Theme 1: Interpreting the data

Interpreting the data comprised a substantial part of the interaction between the facilitator and the physicians in the AGF sessions. This process was always present and consisted of cycles of reactions to the data ranging from satisfaction to skepticism; understanding the data in the report through clarifying the findings and questioning the facilitator; justifying or contextualizing the data by trying to identify potential explanations for the findings relating to personal, patient, system, or other factors; and reflecting on the data by sharing with the facilitator and other group members the findings of their own reports and their personal experiences and practices. Representative quotations for this theme are shown in Table 2.

**Table 2:** Representative quotations about interpreting the data

Sub-theme	
Reactions to the data	Satisfaction: case 2C participant
	“One of my thoughts was … whatever the number is, 5 or 6 people that fall into whichever category. And so now it makes me wonder what did I do there? Cause that 2 from an individual perspective is a very small number. Why did I prescribe and not discontinue? Why did I discontinue and not re-prescribe? Why did I prescribe these drugs rather than those drugs in this particular case? When the absolute number for me as an individual is so small, and I scratch my head about that. So that, it’s fascinating to see where I hit with the rest of everyone else.”
	Skepticism: case 1 participant
	SPEAKER X: “And I’ll just mention all my 10 patients said not recorded and I never do an IV induction and I’m not sure how that data was…”
Understanding and questioning	Case 2A participant*:* “I have a question about that last chart. I am just trying to tease out in my mind where the hospice patients are. If they would pass away post-discharge, so how does that, would they drop from that? I am just wondering, I am trying to sort out…”
	Case 2B: “How did you screen out the group of patients where medications were ordered in your absence, like when the psychiatrist or _____ the orders comes under your name?”
	Case 3 participant: “Is this all procedures, is it, ER?” Moderator: “Yes. So again, … All general surgery Yes, so all of the general surgery, so the total number of patients is 716, so this data, … represents 72% of all procedures because those are the cases that were given Ketorolac and then in the chart below, that’s looking at the 512.” Participant: “How do you tease out what’s a general anesthetic and what’s a spinal?...or a regional…”
Justifying/contextualizing	Findings that physicians attributed to the behavior of others
	Case 1 participant (referring to differences in surgical techniques and surgeons’ practices): “And 25 minutes versus just short of 4 minutes for some of them, as well.” Moderator: “And as well as prescribing when they are home. Some [surgeons] never give morphine. Some will only give, like, Tylenol or something. Some will give oral morphine. So, it’s not surprising pain scores vary.”
	Findings that result from external factors (caring for hospice patients in this example): case 2A
	Moderator: “So again, on the other hand you can see this person out here is probably a better example, saw lots of patients, 35, and half of them got either an antipsychotic or a sedative...” Speaker 1: “... So that’s probably where my midazolam comes from. Because we use tons of midazolam, Haldol, and stuff on hospice.”
	Findings that physicians attributed to personal practices
	Case 2A participant: (discussing why order a zopiclone on admission): “So, cause one of the things … I try to anticipate the calls I am going to have when I am the ward doctor, and try to prevent those. One of those is zopiclone.”
	Case 3 participant (discussing use of ketamine for pain control intra-operatively): “I was using it for a while just to, my numbers are actually very low cause I used it for a while and then I got to know the surgeons and realized that for the most part, I know which ones are really good at [inaudible] filtration and the patients are waking up very comfortable, they are actually not requiring a lot of opioids, so then I did not feel like they needed the Ketamine.”
	Findings that physicians attributed to patient factors
	Case 2A participant: “Let us say when I look at my own it’s the same thing. I see drugs on mine that I am like, I can use that once a year. Like chlorpromazine, I guarantee you it’s because somebody had hiccups from something and I gave them chlorpromazine. I never use that drug.”
	Case 2B participant: “There is a subtly different patient population as well. [Hospital D] is quite a bit older than [Hospital B].”
	Case 2C participant: “I do not know, I do not know if it would, if the numbers would be large enough to have separate graphs for antipsychotics and sedatives. Sedatives like specifically zopiclone, just because probably the populations that would use those 2 medications are different. You know, just for your sleep, difficulty sleeping in hospital, versus dementia with behavioural issues for antipsychotics.”
	Findings that physicians attributed to system factors:
	Case 2A
	Speaker 1 participant “The fall of 2013, was that our Ativan shortage too? Because that will tie in….Because that’ll factor into this, too, because we were ordering goofy things because we could not get the stuff we usually would use. Because that lovely plant in Quebec was down for so long. …Yeah, but midazolam is only IV. So, I used it to substitute for [IV] Ativan in a few people at times.”
	Case 2D participant: “Maybe not that the patients that we see here are different, but our prescribing practices might be influence by like the consultants that we work with in this hospital, or the particular group that we work within.”
Reflecting	Case 1: Participants sharing their practices (discussing the use of nitrous in anesthesia in children): Speaker 1 “[NAME] tried it, without nitrous. I made a switch. Your induction. If that’s your induction try doing it without. Because traditionally we did it when this apoptosis came out I decided to delete nitrous from my practice. So, I have zero for induction. And I haven’t really noticed that longer an induction”. Speaker 2 “I don’t use it either.” Speaker 1 “In fact, I don’t notice a longer induction time actually. So, you might be saving 30 s or something. I haven’t documented it. But it’s something you could try to avoid having to push a button and remembering one more button. Just use air oxygen.”

#### Theme 2: Understanding evidence about best practices

In some of the AGFs, participants commented about specific evidence for best practices, or lack thereof, in relation to the clinical interventions addressed in their AGF reports. Discussion of evidence typically occurred once clarifying questions about the data had been addressed. Identification of certain practice patterns by group members resulted in discussion about relevant evidence and experiences. Project 3 elicited the most evidence-oriented discussion; the project lead had circulated articles to the group prior to the session and there were frequent references and questions about this evidence throughout the session. Examples are shown in Table 3.

**Table 3:** Representative examples of physicians discussing evidence during AGFs

Discussing evidence for best practices	Representative quotations
Case 2B	Participant [raising recent guidelines for the group]. “There’s a bunch of new Choosing Wisely recommendations that came out not last week but the week before. And under the psychiatrists’ [Choosing Wisely Recommendations] there’s a number of them deal with antipsychotics as well, too.”
Case 3	Participant [discussing safety of steroids in diabetic patients]: “And another thing about Dexamethasone… there was a study that showed the glucose jump after single dose of in diabetic is not actually more than, you know, in normal non-diabetic. So, unless it’s a [inaudible] insulin and, you know, with complications, I use it on diabetics too because it’s [inaudible] oral hypoglycemics so it’s, apparently, it’s not affected, like they are not getting the coma after this single dose of Dexamethasone.”

#### Themes 3 and 4: Change cues and change talk

The third major theme emerging from the qualitative analysis was that of “change cues.” This theme is necessarily linked to the final theme, “change talk.” These two themes are presented together because change talk was always preceded by a change cue from a participant, but not all change cues were followed by “change talk,” depending on whether the facilitator acknowledged and promoted discussion on any given change cue. Change cues were defined as turning points in the group discussion, initiated by a brief comment highlighting the importance of a performance gap revealed by the data reports. These were raised spontaneously, usually by a group member rather than the facilitator. In some instances, these cues led immediately to focusing the group discussion on a particular issue that was identified as important. The cues usually consisted of a member pointing out that a specific finding was important and that their group should address it. Typically, these “cues” would lead directly to a discussion of how a care gap could be addressed, what would need to be done, and by whom. We referred to this ensuing discussion as “change talk.” On a few occasions, the research team identified, from the transcripts, a change cue that was *not* followed by change talk. On these occasions, the facilitator, who was not a member of the group, moved on from the cue to the next item in the report without allowing for further discussion about the issue that was raised. Representative examples of change cues and change talk are shown in Table 4.

**Table 4:** Representative examples of change cues and change talk during AGFs

	Representative quotation
Change cue and ensuing change talk	Case 2A participant (discussing data on sedatives and anti-psychotics in the elderly): “… we need support and the region sometimes are not thinking about, so we are all going to have to commit to better, … make sure we are not ordering vitals or meds at night. There’s one. It’s a big one to just keep in our head once in a while, once a week, to just check that.”
	Case 2A: In response to the change cue above, participants offered to take leadership on and identify specific action items to address prescribing of sedatives: Speaker 1: “This is a QI project, I can take that one.” Speaker 2: “I just feel like it will need some sustained attention. Otherwise, it’ll be just one of many CMEs we have had all year. I think it’s important.” Speaker 1: “Then can I hear from this group one list of 3 recommendations that I can take down to the group. Three easy to remember QI things that I can do as a whole and email everybody.”
Change cue and ensuing change talk	A group participant in an anesthesia study identified a priority issue in clear language: Case 1: Speaker 1: “Our biggest problem post-op is pain.” Speaker 2: “Yeah, it is.” Speaker 1: “If you look at the data. So if we can improve intra-operative management by ketamine it would change my practice.” Speaker 2: “Without jeopardizing the nausea and vomiting.”
Change cue without change talk	A group member identifies a key issue and the facilitator moves on to the next topic to explain in the individual data reports. No change talk occurs: Speaker 1: “So this is a really important thing that are resulting in increased usage of sedative, which is really the point, right*.* Use less of them.”
	Facilitator
	“…That’s, I think, the whole idea behind this kind of education, right? Okay, well let us go over the individual. And we can always come back at any time to anything else that needs clarification. So, as I said, we limited docs that got included in this to those that had at least 10 visits…”

**Fig. 2:**
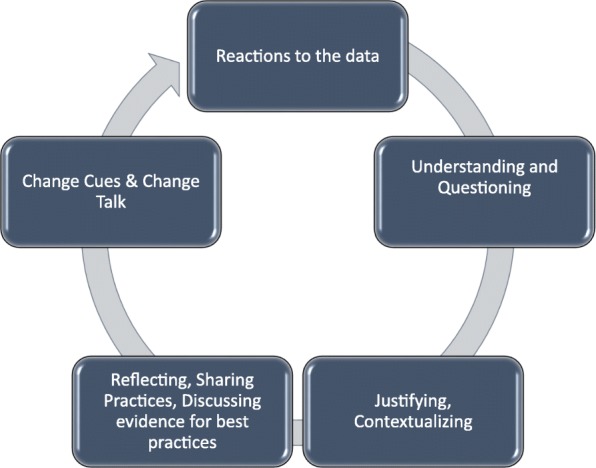
Conceptual model of the cycle of physician behaviors in AGFs. During AGFs, as each new data point was reviewed by the group, physicians progressed through this complement of behaviors, beginning with reactions, then questioning and understanding, and reflection. Opportunities to react to data and address data limitations, skepticism, and surprise would be followed by efforts to understand the AF reports and tables. These steps were necessary pre-requisites to reflection, which included group discussions of individual practice patterns, variations, and experiences as well as guidelines and clinical best practices. Emergent from reflection would be change cues raised by group members. Change cues would routinely pivot the discussion towards action planning. The cycle would repeat with each new data point discussion over the course of the AGF

The results of the thematic analysis were synthesized by the research team to develop a conceptual model to illustrate the cycle of behaviors that typified our AGFs. The model is shown in Fig. 2.

## Discussion

The audit and feedback literature includes many studies demonstrating highly variable design and effectiveness of AF interventions [2, 6]. The relevant literature exists across several disciplines including implementation science, behavioral science, and educational science. Colquhoun and colleagues identified the need to pull together theories from these disciplines to better understand the best way to optimize AF [4, 5].

Better understanding of the nature of physicians’ responses to receiving *feedback* in AF interventions could contribute to improving the effectiveness of AF interventions overall. In this study, we describe physician reactions and responses to a novel form of AF: in-person, facilitated, and group AF sessions. Further, we offer theory-informed hypotheses about how group feedback sessions drive participants towards planning for change.

Our study explored physician behavior in AF interventions that employ best practices from implementation science (relevant, impactful questions, individualized data, and comparators) and the educational feedback literature, namely, in-person, facilitated feedback [7, 15, 26, 27]. Further, we chose to conduct this feedback in a group setting to try to understand the role of socially constructed learning in AF design [13, 14, 28].

We found that a predictable set of behaviors occurred in each of the six audit and feedback projects we studied. There was an iterative process of interpreting the data, discussing relevant best evidence from the literature, and then raising change cues and deciding how to enact change (change talk). The process of interpreting the data was complex and consisted of multiple behaviors that occurred in a predictable sequence for each new data point in the AF presentation: reactions, understanding and questioning, contextualizing, and reflecting. This series of behaviors is consistent with the work of Sargeant et al. who developed the R2C2 model (build *r*elationship, explore *r*eactions, explore *c*ontent, *c*oach for performance change) [15]. Working through these behavioral processes was a pre-requisite to arriving at “change talk” or the performance change aspect of the model. Sargeant et al. highlighted the facilitator’s role in helping participants to navigate through their reactions to data, to understand their data, and then to plan for change [15]. The most successful AGFs in this study were those in which there was strong participation from or co-facilitation with a member of the participant group. A non-group member facilitator, while expert at interpreting the data risked missing relevant change cues and the resultant opportunities to begin action planning.

There was an additional factor identified in this study. Progressing through the cycle hinged not only on the role of the facilitator, but also on the interactions between the members of the group. Indeed, it was most often a spontaneous utterance from a group member (a change cue) that would pivot the direction of conversation towards planning for change. This highlights the added value of using socially constructed learning in the design of feedback sessions for AF [5].

Social learning theory provides the main theoretical basis for using a *group* approach to support learning and behavior change based on AF [13]. Bandura posited that although learning occurs in a variety of ways (trial and error, anticipation of potential consequences or rewards, and others), the ability to learn through observation of and interaction with others is critical to efficient learning and adoption of new behaviors [13]. We observed that it was the interaction *between* group members that most often led to discussing evidence-based practices, raising change cues, and moving to action planning. The process of watching peers, within the group, role model the sharing of and reflection on their own individual data appeared to facilitate the willingness of other group members to do the same. In our sessions, this sharing and reflective behavior generally led to the rise of change cues and change talk during the sessions.

The value of the presence of peers in our AGFs may also be viewed through the lens of the “subjective norms,” described in the theory of planned behavior (TPB) [29]. Ajzen identified subjective norms as an important influence on individuals’ change intentions in the TPB [29]. The subjective norms Ajzen described could play a role in our AGF participants’ willingness to engage in change talk [29]. Ajzen defined subjective norms as “perceived social pressure to engage or not engage in a behaviour” [29]. By creating a situation in which respected peers are encouraged to problem-solve practice gaps and reflect on their individual performance together, we propose that AGF creates such social pressure to engage in change talk.

Several aspects of the findings can also be explained by the AGT and the deliberate choices made by the CPLP. A mastery goal orientation or a focus on competence was promoted through the collective group decision as to which competency area to address accompanied by actionable goals [19]. This was also facilitated by the provision of confidential individual data to allow self-identification of competence [21]. The opportunity to explore the data within a collegial environment [23] ensured understanding of the content which appeared to promote goal setting and a collaborative commitment to change behavior. There has been little exploration in the academic community of the impact of the social context on AGT and the impact on subsequent behavior.

A performance-orientated approach to goal achievement was also considered, because of our use of comparator data in the sessions [20, 22] . However, in this study, the comparison of data between peers did not appear to invoke a competitive approach to problem solving, which is known to be a potential cause of performance avoidant behavior [20, 22]. The findings suggest that promoting a mastery goal orientation contributed to the motivation to improve performance and thereby enhance self-efficacy and the likelihood of goal attainment.

Finally, commitment to change is known to increase physician uptake of new evidence into practice; therefore, change cues and ensuing change talk were considered desirable behaviors in AGFs [30]. Indeed, it has been suggested that commitment to change can be taken as a reasonable marker for practice change [31]. Further, Armson et al. suggested that the cognitive complexity of the change commitments may correlate with increased likelihood of change enactment [31]. In our study, groups of physicians collaboratively explored barriers and enablers to change and identified key steps that would be required to enact change in some of their “change talk.” These activities represent group-level critical reflection, which is a higher order cognitive activity than simply acquiring new knowledge, for example. The group discourse on the interpretation and implications of the data findings and variations in practice engendered deep reflection and knowledge sharing between participants during the sessions. The creation of an implementation plan as a part of the AGFs represents a complex and in-depth form of commitment to change and may therefore account for an additional element of the AGFs process that would be expected to lead to behavior change.

### Limitations and future research

There are several limitations of this study. The principal limitation relates to reproducibility. Our findings of physician behaviors in AGFs represent the collected observations over the course of six AGFs, five hospitals, across three specialties, but included only consenting physicians. The authors acknowledge the risk of selection bias in our participants and that there may be unique system or organizational factors at our center (such as the existence of the CPLP) that facilitated the success of these programs, which may not generalize to AGFs developed in other centers.

## Conclusion

Based on the findings of our study, we offer one primary contribution to the AF literature: a conceptual model that describes the behaviors of physicians who receive performance feedback in a group setting with their peers. This model shows that physicians move through a discrete series of reactions and behaviors during AF in a group setting and that the presence of peers during the sessions leads to change planning.

## Abbreviations

AF: Audit and feedback
AGF: Audit and group feedback
AGFs: Audit and group feedback sessions
CPLP: Calgary Physician Learning Program
TPB: Theory of planned behavior
